# Is device-measured physical activity associated with musculoskeletal disorders among young adult Finnish men?

**DOI:** 10.3389/fspor.2024.1346118

**Published:** 2024-06-04

**Authors:** Lumi Sipilä, Harri Sievänen, Jani Raitanen, Heikki Kyröläinen, Tommi Vasankari, Jani P. Vaara, Tuomas Honkanen

**Affiliations:** ^1^ Faculty of Medicine, University of Helsinki, Helsinki, Finland; ^2^ The UKK Institute for Health Promotion Research, Tampere, Finland; ^3^ Faculty of Social Sciences (Health Sciences), Tampere University, Tampere, Finland; ^4^ Department of Leadership and Military Pedagogy, National Defence University, Helsinki, Finland; ^5^ Faculty of Sport and Health Sciences, University of Jyväskylä, Jyväskylä, Finland; ^6^ Faculty of Medicine and Health Technology, Tampere University, Tampere, Finland; ^7^ Aeromedical Centre, Centre for Military Medicine, Helsinki, Finland

**Keywords:** physical activity, musculoskeletal disorders, fitness, accelerometer, young men

## Abstract

**Background:**

Musculoskeletal (MSK) disorders represent a significant burden to society and can be unpleasant for the affected individuals. Physical activity (PA) can prevent MSK disorders while conferring other health benefits. The present study aimed to investigate associations between device-measured PA and perceived MSK disorders among young adult men.

**Methods:**

PA at different intensity levels, standing, and sedentary behavior were measured with a hip-worn accelerometer in a cohort of 422 young adult Finnish men aged 26 years on average. The incidences of three common MSK disorders (viz., knee pain, lumbar radicular pain, and lumbago pain) during the last month were inquired by a questionnaire. Binary logistic regression was used to examine the associations between the MSK outcomes and explanatory PA variables (PA times at different intensity levels, standing, and sedentary times). The models were controlled for age, education, smoking, BMI, and maximal oxygen uptake.

**Results:**

PA, standing, and sedentary times were not significantly associated with the incidence of perceived MSK pain during the last month, except for lumbago pain. Lumbago pain was slightly more probable if the time spent in light PA increased, even after controlling for potential confounding factors, including moderate-to-vigorous PA, with an odds ratio (OR) of 1.07 (95% CI: 1.02–1.14). Sedentary time showed an opposite association, with an OR of 0.98 (95% CI: 0.96–1.00).

**Conclusions:**

There were neither positive nor negative clinically meaningful associations between PA and recent MSK disorders among young adult men. The result is surprising and requires further confirmation.

## 1 Introduction

Musculoskeletal (MSK) disorders are conditions that adversely affect joints, muscles, tendons, and other tissues of the MSK system. The Global Burden of Disease categorizes osteoarthritis, rheumatoid arthritis, gout, low back pain, and neck pain as MSK disorders, whereas other conditions, such as knee pain are classified under a residual MSK category (1).

Physical activity (PA) and exercise have been reported to reduce inflammation in the body and alleviate MSK disorders, presumably through modulating pro- and anti-inflammatory cytokines (2). MSK pain may have a big impact not only on a person's quality of life but also on society, manifesting as a huge economic burden. The costs of MSK (and other) disorders comprise three different components: direct, indirect, and intangible costs. Direct costs include prevention, treatment, and all medical care. Indirect costs include lost work due to sick leaves and consequently lost productivity. Intangible costs include psychosocial factors such as reduced quality of life and increased amount of stress (3).

Besides PA, other lifestyle-related factors such as smoking and overweight also count. Smoking accentuates MSK disorders by aggravating symptoms, causing disease progression, and contributing to comorbidities (4). People who smoke or have a history of smoking have a higher prevalence and incidence of lower back pain and lumbar radicular pain (5, 6). However, earlier studies on the association between smoking and the amount of PA have remained inconclusive (7). Overweight or high body mass index (BMI) increases the risk for MSK disorders (8). Several studies have shown that people with high BMI are physically less active than people with normal weight (9).

Compared to commonly used questionnaires, accelerometers offer advantages in measuring PA such as their accuracy and capacity to continuously collect real-time data on PA and sedentary behavior (SB) without subjective influence (10). Accelerometer-measured moderate-to-vigorous PA (MVPA) is associated with broader knees in middle-aged Finns, while larger joints may contribute to fewer knee problems (11). Accelerometer-based light, vigorous, and very vigorous PA is associated with more lumbar radicular pain and back static muscular endurance (12). Accelerometer-measured PA is also associated with fewer shoulder and ankle problems among women aged 50–64 years (13). In middle-aged women, more PA is associated with better MSK health in general (14). The majority of earlier studies based on accelerometer-measured PA seem to be conducted in middle-aged populations. However, this age-specific knowledge is not generalizable to young people, and more information on this topic is needed. According to a recent population-based study, the prevalence of MSK problems is 47.7% for back pain among men aged 18–29 years (15).

The rationale of the present study of young adult men is that MSK disorders could be prevented by knowing the major modifiable factors that contribute to their incidence. Young adult men are a relatively healthy group and represent a hard-to-reach target group in research. The present study aimed to investigate the associations between device-measured PA and common MSK disorders (lower back pain, lumbago pain, and knee pain) among young adult Finnish men. We hypothesize that more daily MVPA is associated with a lower probability of recently perceived MSK disorders.

## 2 Materials and methods

### 2.1 Participants

The participants comprised 777 young adult Finnish men with a mean age of 26 years (SD, 7), who participated in mandatory military refresher training by the Finnish Defence Forces. The data were collected during seven different refresher courses between May and November 2015. Participation in the study was voluntary. Among the 777 participants, 355 were excluded from the final study sample due to missing accelerometer data. The final sample size was 421 because one person had missing data on the pain variables.

The study was approved by the ethical committees of the University of Jyväskylä and the Central Finnish Health Care District, as well as the Defence Command of the Finnish Defence Forces (AM5527). Before attending the study, written informed consent was obtained from all participants.

### 2.2 Assessment of PA and physical fitness

Participants' PA times at different intensities, standing, and sedentary times were measured using a triaxial accelerometer with a sampling frequency of 100 Hz (Hookie AM 20/30, Traxmeet Oy, Finland). The accelerometer data were collected in the following week after the military refresher course in everyday life at home. For valid data, the accelerometer had to be used for at least 4 days, 10 h per day at minimum. The accelerometer was kept on the right side of the hip in an elastic belt and was not worn during the nighttime.

The raw triaxial accelerometer data were analyzed with validated mean amplitude deviation (MAD) and angle for posture estimation (APE) methods (16, 17). The epoch length was 6 s. The MAD and APE analysis methods provide accurate information on PA performed at different intensities and SB in terms of standing, sitting, and lying. The acceleration data were converted to metabolic equivalent (MET) values using a validated method (15). The MET value represents the energy expenditure during the PA level of interest compared to energy expenditure at rest. The MET values were categorized into different ranges, where SB, including standing, denotes energy expenditure at a level of <1.5 MET; light physical activity (LPA), 1.5–3 MET; moderate physical activity (MPA), 3–6 MET; and vigorous physical activity (VPA), >6 MET. If the continuous quiescent (i.e., not collecting data) time of the accelerometer was longer than 120 min, the given period was considered non-wear time (18).

Participants' cardiorespiratory fitness was evaluated with a bicycle ergometer test and defined as maximal oxygen uptake (VO_2max_) (19). The initial workload of the test was 50 W and was increased to 25 W every 2 min until volitional exhaustion. VO_2max_ was estimated using the maximal heart rate and maximal power (W) (Fitware, Mikkeli, Finland) with the following equation: VO_2max_ (ml·kg^−1^·min^−1^) = 12.35 × Pmax/body weight (kg) + 3.5, where Pmax is the maximal power attained in the test.

### 2.3 Pain and disability questionnaire

Perceived MSK disorders were inquired about by a questionnaire. Three different questions about MSK disorders were asked: (1) “How often have you had lumbar radicular pain in the last month?”, (2) “How often have you had lumbago pain in the last month?”, and (3) “How often have you had knee pain in the last month?”. As our study was part of the Finnish Reservist 2015 study, certain MSK disorders were already chosen in the original study setting. An illustration was provided to clarify the lumbar region. All three questions had the same answering alternatives: (a) none, (b) 1–7 days, (c) 8–14 days, (d) over 14 days but not daily, and (e) daily. The lumbar radicular pain was considered to radiate under the knee. The lumbago pain was considered a sudden pain in the area illustrated in the picture of the lumbar region and gluteal muscles. A 100 mm visual analog scale was used to describe the severity of back pain and lower limb pain during the last 7 days: 0 denoted no pain, and 100 denoted the worst possible imaginable pain. The same questionnaire was used in an earlier study (20).

### 2.4 Statistical analysis

Descriptive characteristics were reported as means and SD or frequencies and percentages. Lying and sitting (i.e., sedentary time), standing, and light, moderate, and vigorous PA times were reported as percentages of the daily accelerometer wear time.

A binary logistic regression analysis was used to examine the associations between the binary outcomes (0 stands for no MSK pain during the last month, and 1 stands for pain at least 1 day during the last month) and continuous explanatory variables (relative times of SB, standing, and PA at different intensity levels). The physical behavior variables in the group not perceiving the pain of interest during the last month served as the reference data. For the analysis, MPA and VPA times were combined, providing MVPA time as a result. Age (continuous), education (categorical), and smoking (categorical) were examined as potential confounding factors in the first adjusted model, and BMI (continuous) and VO_2max_ (continuous) were added to the second adjustment. The multicollinearity between explanatory variables was tested by calculating the variance inflation factor, and no evidence of collinearity was observed.

Odds ratios (OR) with 95% CI were calculated for both unadjusted and adjusted models. As to the interpretation of the OR, with every 1% increase in the explanatory variable (e.g., SB time), the odds of having the pain of interest are multiplied by the OR.

A *p*-value of <0.05 was considered statistically significant. The data were analyzed with Stata Version 15.1 (College Station, TX, USA: StataCorp LLC).

## 3 Results

The descriptive background data of the participants, perceived MSK disorders, and the device-measured PA variables are presented in Table 1. Of the mean daily accelerometer wear time, the participants mostly sat (52.5%). Sedentary time combining sitting and lying represented 65.4% of the measurement time. On average, the participants stood at 11.9% and were engaged in LPA at 13.5% and in MVPA at 9.2% of their daily time. The proportion of VPA was 0.5%. Both relative (in %) and absolute times (in minutes) spent in different physical behaviors in the three MSK groups are presented in Table 2.

**Table 1:** Descriptive background data of the participants (*n* = 422).

	Mean (SD) or *n* (%)	Missing *n*
Clinical background variables		
Age (years)	28.5 (7.3)	–
Body mass (kg)	82.2 (15.1)	8
Height (cm)	179.4 (6.3)	7
BMI (kg/m^2^)	25.5 (4.1)	8
Fat (%)	17.6 (7.8)	7
Waist circumference (cm)	88.4 (11.1)	7
Maximal oxygen uptake (ml/kg/min)	41.3 (7.9)	9
Education		2
Primary/vocational school	185 (44.0)	
High school/institute/university of applied sciences/academic degree	235 (56.0)	
Smoking		
Never smoked regularly	216 (51.3)	
Quitted smoking >6 months ago	83 (19.7)	
Quitted smoking <6 months ago	24 (5.7)	
Regular smoking	98 (23.3)	
Attendance in competitive sports at the age of 12 years		1
None	149 (35.4)	
School	30 (7.1)	
Company	100 (23.8)	
Regional level	108 (25.7)	
National level	34 (8.1)	
Incidence of lumbar radicular pain during the last month	1	
None	328 (77.9)	
1–7 days	82 (19.5)	
8–14 days	6 (1.4)	
Over 14 days, but not daily	2 (0.5)	
Daily	3 (0.7)	
Incidence of lumbago pain during the last month	1	
None	302 (71.7)	
1–7 days	105 (24.9)	
8–14 days	6 (1.4)	
Over 14 days, but not daily	5 (1.2)	
Daily	3 (0.7)	
Incidence of knee pain during the last month	1	
None	284 (67.5)	
1–7 days	118 (28.0)	
8–14 days	11 (2.6)	
Over 14 days, but not daily	4 (1.0)	
Daily	4 (1.0)	

The number of missing data is indicated in the right column.

**Table 2:** Mean (SD) relative (%) and absolute (min) device-measured SB, standing, LPA, and MVPA times of the daily wear time broken down by the MSK pain groups.

MSK pain	Lumbar radicular pain		Lumbago pain		Knee pain	
	No	Yes	No	Yes	No	Yes
*N*	328	93	302	119	284	137
SB (%)	65.6 (11.1)	64.7 (10.6)	66.0 (10.9)	63.8 (11.2)	65.3 (11.1)	65.6 (11.0)
SB (min)	601 (145)	592 (143)	607 (146)	579 (139)	597 (141)	604 (151)
Standing (%)	11.9 (5.0)	12.0 (4.6)	11.7 (4.9)	12.4 (5.0)	11.9 (5.2)	11.9 (4.4)
Standing (min)	107 (45)	109 (41)	106 (44)	112 (46)	108 (47)	107 (39)
LPA (%)	13.3 (5.1)	13.9 (5.2)	13.3 (5.1)	14.4 (5.1)	13.4 (5.1)	13.5 (5.3)
LPA (min)	121 (48)	125 (45)	119 (47)	130 (48)	122 (48)	121 (47)
MVPA (%)	9.2 (3.8)	9.3 (3.6)	9.1 (3.7)	9.4 (3.9)	9.3 (3.8)	9.0 (3.7)
MVPA (min)	83 (34)	84 (33)	84 (35)	84 (35)	84 (35)	81 (32)

The binary logistic regression analysis did not reveal any significant association between physical behavior and the incidence of perceived MSK pain during the last month, except for the significant association (OR: 1.05, 95% CI: 1.01–1.09, *p* < 0.05) between LPA and lumbago pain (Table 3). Controlling for the confounding factors gave similar results (Models 2 and 3). Sedentary time showed a significant, but opposite, association (OR: 0.98, 95% CI: 0.96–1.00, *p* < 0.05) with lumbago pain. The robustness of the observed associations was further tested by including MPVA time as an additional confounding factor, but its influence remained negligible (Table 4).

**Table 3:** Associations between physical behavior and incidence of perceived MSK pains during the last month.

MSK outcome	Model 1	Model 2	Model 3
	OR (95% CI)	OR (95% CI)	OR (95% CI)
*n*	421	420	403
Lumbar radicular pain			
Sedentary time	0.99 (0.97–1.01)	0.99 (0.97–1.02)	1.00 (0.97–1.02)
Standing	1.01 (0.96–1.05)	1.00 (0.96–1.05)	1.01 (0.95–1.06)
Light activity	1.02 (0.98–1.07)	1.02 (0.97–1.07)	1.01 (0.96–1.06)
Moderate-to-vigorous activity	1.01 (0.95–1.07)	1.01 (0.95–1.07)	1.00 (0.93–1.07)
Lumbago pain			
Sedentary time	0.98 (0.96–1.00)	0.98 (0.96–1.00)	0.98 (0.96–1.00)*
Standing	1.03 (0.99–1.07)	1.03 (0.99–1.08)	1.03 (0.98–1.08)
Light activity	1.05 (1.01–1.09)*	1.05 (1.01–1.10)*	1.06 (1.01–1.11)*
Moderate-to-vigorous activity	1.02 (0.96–1.08)	1.01 (0.96–1.07)	1.02 (0.96–1.09)
Knee pain			
Sedentary time	1.00 (0.99–1.02)	1.00 (0.98–1.02)	1.00 (0.98–1.02)
Standing	1.00 (0.96–1.04)	1.01 (0.96–1.05)	1.01 (0.96–1.06)
Light activity	1.00 (0.96–1.04)	1.00 (0.96–1.04)	1.00 (0.96–1.04)
Moderate-to-vigorous activity	0.98 (0.92–1.03)	0.97 (0.92–1.03)	0.97 (0.92–1.03)

Explanatory PA variables were served to the model as relative (%) values of the daily accelerometer wear time.

Model 1 = unadjusted model.

Model 2 = adjusted for age, education, and smoking.

Model 3 = adjusted for age, education, smoking, BMI, and VO_2max_.

* *p* < 0.05.

**Table 4:** Association between physical behavior and incidence of perceived MSK pains during the last month controlled for MPVA time.

MSK outcome	Model 1	Model 2	Model 3
	OR (95% CI)	OR (95% CI)	OR (95% CI)
*n*	421	420	403
Lumbar radicular pain			
Sedentary time	0.99 (0.96–1.02)	0.99 (0.96–1.03)	0.99 (0.96–1.03)
Standing	1.01 (0.96–1.06)	1.00 (0.95–1.05)	1.01 (0.95–1.06)
Light activity	1.03 (0.97–1.09)	1.02 (0.96–1.09)	1.02 (0.96–1.09)
Lumbago pain			
Sedentary time	0.97 (0.94–1.00)*	0.96 (0.93–1.00)*	0.97 (0.93–1.00)*
Standing	1.03 (0.98–1.07)	1.03 (0.98–1.08)	1.03 (0.98–1.08)
Light activity	1.07 (1.01–1.12)*	1.07 (1.02–1.13)*	1.07 (1.02–1.14)*
Knee pain			
Sedentary time	0.99 (0.96–1.02)	0.99 (0.96–1.02)	0.99 (0.96–1.02)
Standing	1.01 (0.96–1.05)	1.01 (0.97–1.06)	1.02 (0.97–1.06)
Light activity	1.02 (0.97–1.07)	1.02 (0.97–1.08)	1.02 (0.96–1.08)

Explanatory PA variables were served to the model as relative (%) values of the daily accelerometer wear time.

Model 1 = adjusted for MVPA.

Model 2 = adjusted for MVPA, age, education, and smoking.

Model 3 = adjusted for MVPA, age, education, smoking, BMI, and VO_2max_.

* *p* < 0.05.

## 4 Discussion

This study investigated the association between accelerometer-measured sedentary time, standing, and PA with perceived MSK disorders among young adult Finnish men. Against our expectations, neither of the device-measured PA, standing, nor sedentary time were significantly associated with the incidence of perceived MSK pain in the last month. Only more relative time spent in LPA was associated with a slightly higher probability for lumbago pain, whereas sedentary time showed the opposite association. Despite being statistically significant, these associations were considered clinically meaningless, because the observed ORs were generally small and indicated that the physical behavior between the young men having and having not perceived lumbago pain during the last month was largely comparable. Further, no significant associations between physical behavior and knee pain or lumbar radicular pain were observed.

Interestingly, our result conflicts with the findings of previous questionnaire-assessed studies (21, 22), suggesting a protective association between PA and MSK disorders. A similar protective association has been observed between accelerometer-assessed PA and different MSK disorders (11, 13, 14). Only accelerometer-based LPA, VPA, and very vigorous PA seems to cause more radicular lumbar pain (12). However, age ranges in other studies (11–14, 21, 22) were wider comprising all-aged adult men and women compared to the present sample of young adult men. In the present study, we found no statistically significant associations between PA and radicular lumbar pain. The fact that PA in some studies was based on subjective questionnaire data may also explain why the results from other studies conflict with our findings. Different PA assessment methods are not comparable or interchangeable, and if a comparison is done, it needs to be done cautiously (23).

Interestingly, more moderate leisure time PA assessed with a questionnaire was associated with a higher probability of lower back pain and neck pain among young adult Finnish men, whereas more moderate-to-high leisure time PA was associated with a higher probability of lumbago pain (20). These findings are in apparent conflict with our observation, suggesting an association between a higher proportion of LPA and perceived lumbago pain. However, our study does not differentiate different kinds of PA domains, i.e., leisure time PA from occupational PA. Leisure time PA has been found more beneficial for health than occupational PA (24).

We found a statistically significant but weak association between LPA and lumbago pain. Among those with lumbago pain during the last month, LPA occupied 130 min (14.4%) of the mean daily accelerometer wear time, whereas among those not reporting lumbago pain, the corresponding time was about 119 min (13.1%). As the accelerometer was worn at least 10 h per day, a 1.3% difference in relative wear time represents only some minutes of the participants’ waking hours, and the clinical meaningfulness of the found association seems marginal. A few minutes in LPA would hardly make a true difference in the incidence of lumbago pain. Since the present study is cross-sectional by design, no causal inference could be made. More LPA and consequent lack of more intensive PA may increase the risk of lumbago pain, or lumbago pain may be the reason for more time spent in LPA because the perceived pain restricts more intensive PA. In our study, the incidence of MSK disorders was inquired before PA was measured. Therefore, recurrent pain, if occurring at the time of PA measurement, could have led to being less physically active compared to habitual PA. On the other hand, the significant association between LPA and lumbago pain can just be a coincidence due to multiple statistical tests. The actual finding of the present study is likely that there is no clinically meaningful association between measured habitual PA and recently perceived MSK disorders in young adult men.

Besides the validated accurate method to measure PA and SB (16, 17, 25), the strength of the present study is the moderate-sized study sample that can be considered a representative sample of healthy young adult men able to participate in a military refresher course. The conscription is defined in the constitution of Finland, and therefore the military service and the following refresher courses are mandatory for men. The proportion of missing data was generally marginal (no more than 2% in a few variables), which hardly confounded the present findings.

Our study has also limitations that need to be considered. The study sample consisted only of young adult men, whereas earlier studies have shown differences in MSK disorders between both sexes. Women tend to have more frequently severe MSK disorders (26). The burden of MSK disorders increases with aging (27). Our relatively young study population might not yet be suffering from remarkable MSK disorders at all. As the data were collected from persons who attended a military refresher course, selection bias is possible and needs to be considered. However, the sample is quite well representative as more than 70% of the conscripts in Finland completed the military service. The reasons for exempting the military service are mainly mental and social instead of MSK disorders (28). Since the participants were aware that their PA was monitored with accelerometers, they might have increased their PA. Also, the hip-worn accelerometer can neither capture all PA involved in resistance or gym training nor evaluate their intensity. Any substantial deviations from habitual physical behavior may have confounded the associations to some extent. However, compared to the PA data of the previous population of similarly aged men (18, 29), the observations are quite comparable. It is also possible that the week after a strenuous military refresher course was not as physically active as usual.

The MSK disorders in this study (knee pain, lumbago pain, and lumbar radicular pain) were reported with a subjective questionnaire, which is a widely used method (20–22) but subject to uncertainties. The participants had to recall the days they had a certain MSK pain during the last month. We did not take into consideration sick leaves, doctors' appointments, or diagnoses due to these MSK disorders. We did not have access to the participants' medical patient records or health register data. Therefore, the earlier injury background is missing, which can be considered a limitation of this study. Reporting the pain variables is inherently highly participant-dependent and cannot be bound to the later days the accelerometer measurements were conducted. A more reliable way to evaluate MSK disorders prospectively would be needed, while the PA measurements should describe each participant's typical habitual PA rather than potential exceptions in physical behavior. The lack of long follow-up of habitual PA is also a limitation of the present study.

Despite the above-discussed limitations, this study brings us relevant information since the present results challenge the notions from earlier studies relying on subjectively assessed PA. In sum, there seems not to be clinically meaningful associations between the device-measured PA and MSK disorders among generally healthy young adult Finnish men. Further research is needed to reveal modifiable behavioral factors that may be utilized in the optimal prevention of MSK disorders. This information is especially important among young people to manage premature disabilities.

## 5 Conclusions

We did not find clinically meaningful associations between accelerometer-measured PA and MSK disorders in young adult Finnish men. The result is surprising as it conflicts with earlier findings on this topic, however, mostly evaluated in middle-aged populations. It may also be so that, in young men, there is no clinically meaningful association between measured PA and recently perceived MSK disorders. Furthermore, more studies with appropriate methods preferably in a prospective setting are required to investigate the causal factors underlying MSK disorders among young men.

## Data Availability

The data analyzed in this study is subject to the following licenses/restrictions: The dataset is owned by the Finnish Defence Forces, which do not share the data. Requests to access these datasets should be directed to jani.raitanen@ukkinstituutti.fi.
